# P2Y Receptors in Bone - Anabolic, Catabolic, or Both?

**DOI:** 10.3389/fendo.2021.818499

**Published:** 2022-01-07

**Authors:** Yuhan Zhou, Hector M. Arredondo, Ning Wang

**Affiliations:** The Mellanby Centre for Musculoskeletal Research, Department of Oncology and Metabolism, The University of Sheffield, Sheffield, United Kingdom

**Keywords:** P2Y receptor, ATP, ADP, osteoblast, osteoclast

## Abstract

P2Y receptors, including eight subtypes, are G protein-coupled receptors that can be activated by extracellular nucleotides. Nearly all P2Y receptors are expressed in bone cells, suggesting their involvements in bone physiology and pathology. However, their exact roles in bone homeostasis are not entirely clear. Therefore, this mini review summarizes new research developments regarding individual P2Y receptors and their roles in bone biology, particularly detailing those which execute both anabolic and catabolic functions. This dual function has highlighted the conundrum of pharmacologically targeting these P2Y receptors in bone-wasting diseases. Further research in finding more precise targeting strategy, such as promoting anabolic effects *via* combining with physical exercise, should be prioritized.

## Introduction

Extracellular nucleotides, whether they are adenosine 5′-triphosphate (ATP), uridine 5’-triphosphate (UTP), or their hydrolysate adenosine adenosine 5′-diphosphate (ADP), uridine-5′-diphosphate (UDP) and UDP-sugars, have been identified as an essential class of signal molecules which can activate the purinergic signalling in diverse types of cells and tissues, thereby mediating biological events (1). Currently, extracellular nucleotides have been found to play key roles in a variety of physiological processes such as tissue homeostasis, wound healing, neurodegeneration, immunity, inflammation, and tumor metastasis (2). Importantly in bone, P2 receptors, purinergic receptors that bind nucleotides, can finely regulate bone cell physiology and modulate bone remodeling (3).

It was not until 1985 that P2 receptors were firstly distinguished into two purinoceptor subtypes based on their pharmacological characteristics: P2X and P2Y receptors (4), followed by further new subtypes such as P2T, P2Z, and P2U receptors (5, 6). In the following decade, to provide a more organized basis, P2 receptors had been re-categorized based on their transduction mechanisms and topology into two major families: the P2X family of ligand-gated ion channel receptors and the P2Y family of G protein-coupled receptors (GPCRs) (7).

To date, seven P2X receptor (P2XR1-7) members have been identified in human. As ligand-gated ion channel receptors, their physiological agonists are mainly ATP. P2XRs were shown to form functional trimers with three ATP binding sites, all of which need to be occupied to trigger the channel opening and subsequently mediate selective permeability to cations, such as Ca^2+^, Na^+^, K^+^ (8, 9). In contrast, the GPCR P2YRs can be activated by adenine and uridine nucleotides. GPCRs have been found as therapeutic targets for a variety of human diseases and more than 40% of modern drugs are GPCRs based (10). Herein, we focus on the recent advances in our understanding of the involvement of P2Y receptors (P2YRs) in bone physiology and pathology.

## P2Y Receptors

**Figure 1:**
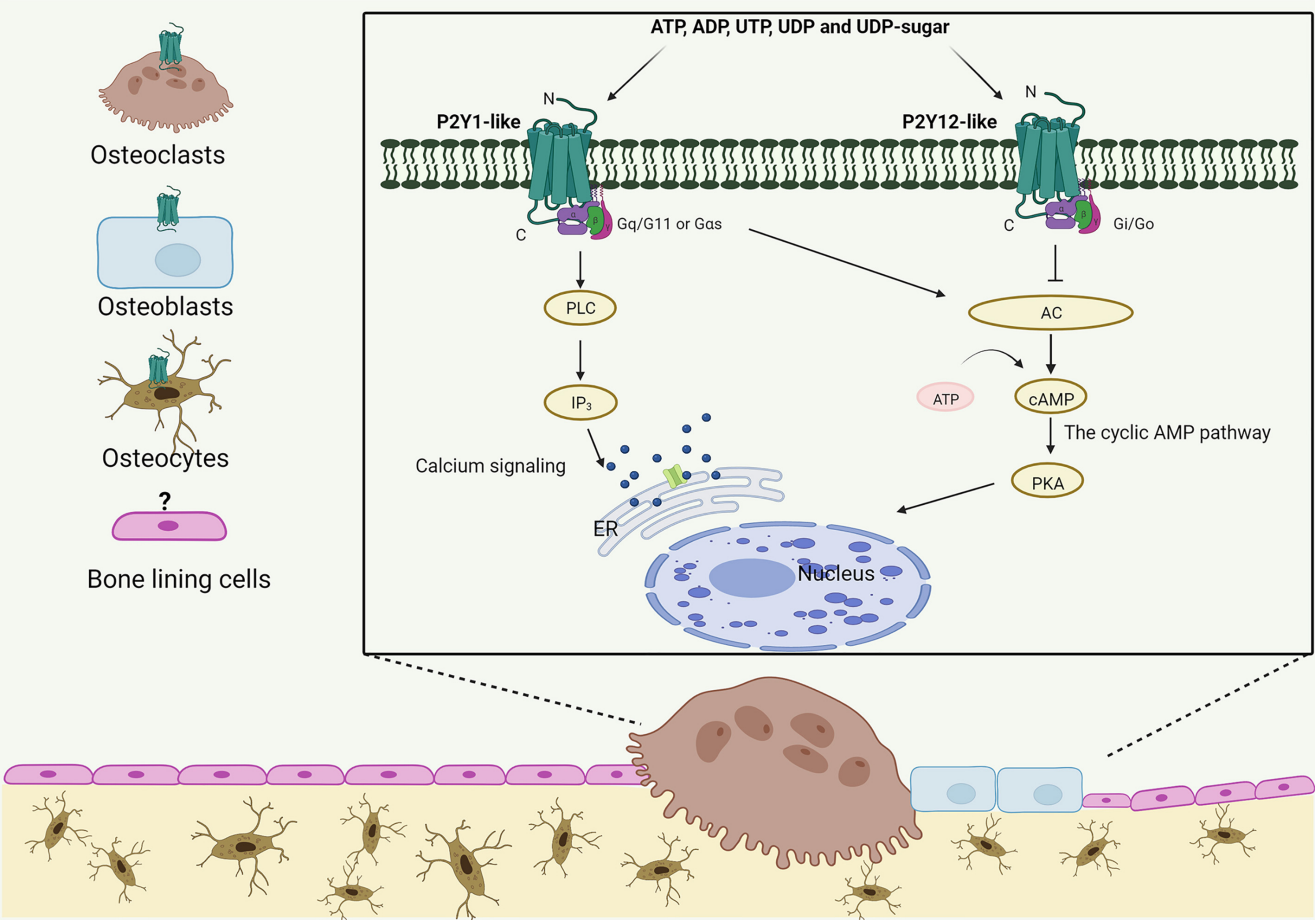
The structure and signaling pathway of P2YRs in bone. P2Y1-like and P2Y12-like receptors are expressed in most types of bone cells (expression status of P2YRs in bone lining cells is current unknown) and are activated by extracellular nucleotides present in the microenvironment. The topological characteristics of P2YRs are: an extracellular N-terminus and an intracellular C-terminus; seven transmembrane domains forming the ligand docking pocket; the C-terminus has the protein kinases binding motif and influence the degree of P2Y subtypes coupling with Gq/G11, Gαs and Gi/Go proteins *via* intracellular loop structural diversity. Binding of different G proteins leads to downstream signaling cascades including calcium signaling transduction and regulation of AC/cAMP pathway (Figure was created with Biorender).

There are eight members of P2YRs and can be divided into two subgroups based on their structure similarities: P2Y1-like GPCRs including P2Y1, P2Y2, P2Y4, P2Y6 and P2Y11; and P2Y12-like GPCRs including P2Y12, P2Y13 and P2Y14. In addition, there are also two non-mammalian p2y8 and tp2y receptors (11). ADP, ATP, UDP, UTP and UDP-sugars have all been identified as agonists for P2YR (12). P2YRs are located on the cell membrane and consists of an extracellular N-terminus, an intracellular C-terminus and seven hydrophobic transmembrane helices connecting three intracellular and extracellular loops. Signaling *via* diverse G proteins activates or inhibits different pathways. The Gq alpha subunit (Gq/11proteins) and Gs alpha subunit (Gαs proteins) are stimulated through binding of extracellular nucleotides to P2Y1-like GPCRs, thereby leading to downstream signal cascade: the activation of phospholipase C/inositol 1,4,5-trisphosphate (PLC/IP_3_) and adenylyl cyclase (AC) pathway, respectively. Compared with P2Y1-like GPCRs, P2Y12-like GPCRs bind to Gi protein alpha subunit (Gi/Go proteins) inhibiting AC pathway resulting in decreased accumulation of cyclic adenosine monophosphate (cAMP) and cAMP-dependent protein kinase (PKA) (**Figure 1**) (13). P2YRs can form multimers in certain circumstances as P2XRs do, but their peptides show lower sequence homology (19-55%) compared to P2XR. Thus, P2YRs have a greater diversity in their pharmacological and operational profiles (8, 14).

## P2YRs in Bone

Bone is a specialized connective tissue that provides internal support in all higher vertebrates. It makes up the skeletal system, together with cartilage (15, 16). In bone, extracellular matrix and cells are the fundamental constituents. The organic matrix is mainly composed of Type I collagen. This matrix is strengthened by inorganic component, mainly carbonated hydroxyapatite. Bone is composed of four different cell types: osteoblast, osteoclast, osteocyte and bone lining cells. Those bone cells together play essential roles in the process of bone modeling and remodeling (16).

The investigation of the role of P2YRs in bone cells can be traced back to early 1990s. Evidence was found for the presence of P2 purinoceptors on both rat and human osteoblast-like cells (17, 18), and P2YRs were shown to mediate the non-selective cation channels and Ca^2+^ dependent K^+^ channels in rat osteoclasts (19). Since then, an increasing number of purinoceptor subtypes were found existing in human bone related cells, by means of immunocytochemistry, *in situ* hybridization, and RT-PCR (20). In the bone microenvironment, nucleotides are intracellularly presented at a normal concentration of 2-5mM but released into extracellular under mechanical stimulation, tissue injury or inflammation situations (21). These extracellular nucleotides are then able to regulate bone remodeling as extracellular signaling molecules, such as functioning as mitogens for osteoblasts and osteoclasts, *via* binding to P2 receptors. Activation of P2Y receptors will couple to downstream signal transduction cascades, including cAMP/PKA pathway and/or IP_3_ hydrolysis by PLC and subsequent release of Ca^2+^ from endoplasmic reticulum (ER) (3). The high concentration of Ca^2+^ will induce the activation of the mitogen-activated protein kinase (MAPK) pathway *via* phosphorylation of extracellular signal-regulated kinase (ERK), especially ERK 1/2 (22).

However, due to the variability of P2YR subtypes, different subtypes play different roles during bone homeostasis, which correlate to their native agonists, binding G protein type, and expression in different bone cells (**Table 1**). Based on the last comprehensive review by Orriss et al. in 2012 (20), we will next review most recent research progress in roles of each individual P2YR subtypes in bone cell biology and bone homeostasis.

**Table 1:** Distribution and characteristics of P2 receptors in bone.

Receptor	Native Agonists	Binding G protein type	Main distribution in bone	Functional effects
P2Y1	ADP>ATP	Gq/11	Osteoblasts and osteoclasts	Enhance PTH-induced Ca^2+^ signaling and ultrasound-induced osteoblast proliferation (3, 23–25). Increase the osteoclast formation and resorption (26). Involve in the mechanotransduction (27).
P2Y2	UTP=>ATP	Gq/11	Osteoblasts, osteoclasts and osteocytes.	Inhibit differentiation and mineralization of osteoblasts (28–32). Induce bone resorption from osteoclasts (32). Propagation of intercellular Ca^2+^ waves (33) and involve in mechanotransduction (27, 34).
P2Y4	UTP=>ATP	Gq/11	Osteoblasts and osteocytes.	Play a ‘off-switch’ role during bone formation with P2Y2R (35).
P2Y6	UDP>UTP>>ATP	Gq/11	Osteoblasts, osteoclasts and osteocytes.	Contribute to osteoclast resorption (36) and enhance osteoclast survival (37). Induce osteogenic differentiation from BMSCs (38). Involve in mechanotransduction (27).
P2Y11	ATP/UTP	Gq/11 and Gαs	Osteoclasts	Inhibit cell migration and bone metastasis in breast cancer (39).
P2Y12	ADP>>ATP	Gi/Go	Osteoblasts, osteoclasts and osteocytes.	Induce osteoblastogenesis and avoid osteoclasts formation (40, 41) but others suggested maintaining osteoclasts activity (42).
P2Y13	ADP>>ATP	Gi/Go	Osteoblasts, osteoclasts and osteocytes.	Induce osteoblasts differentiation and osteoclastogenesis (43–47). involve in mechanotransduction (27).
P2Y14	UDP-sugar/UDP	Gi/Go	Osteoblasts and osteoclasts	Promote osteoclastogenesis (48, 49). Promote osteoblast proliferation (50). Involved in mechanotransduction (27).

### P2Y1-Like Receptors

The Gq/11-coupled P2Y1 receptor is predominantly activated by ADP, whilst ATP can act as its antagonist or partial agonist (51). P2Y1Rs were initially found to be mainly involved in the regulation of osteoclast-mediated bone loss. 2-MethylthioADP (2-MeSADP) was developed as a highly selective P2Y1R agonist which mimic ADP and lead to P2Y1R-mediated cell responses (52). This agonist can stimulate osteoclast formation and resorptive activity at a concentration of only 0.1-10 μM (26). Meanwhile, P2Y1Rs were found to be expressed at transcriptional and translational levels in both osteoblasts and osteoclasts. P2Y1R is reported to modulate osteoblastic responses to systemic factors such as parathyroid hormone (PTH) through up-regulating the phosphorylation of cAMP response element-binding protein and c-fos expression (23). ATP acts as the locally released costimulant to enhance Ca^2+^ release in response to PTH, through activation of P2Y1R (3, 23). Besides, another study suggested that P2Y1R was stimulated by ATP released from osteoblasts after ultrasound induction, which acts in an autocrine manner to induce osteoblastogenesis (24). Further *in vivo* studies underscored the important role of P2Y1R in osteoblastic biology owing to the availability of P2Y1 knockout (KO) mice. Two-month-old global P2Y1R KO mice showed 5-14% decrease in bone mineral density (BMD) and bone mineral content (BMC) determined using DEXA scan. Significant decreases in trabecular parameters were detected through Micro-CT analysis (25). These findings indicate that, although predominantly plays a pro-osteogenic role, the P2Y1R may also regulate osteoclast differentiation.

In contrast to P2Y1R, P2Y2R has initially been suggested to play an inhibitory role in bone mineralization (28), as the activation of P2Y2R was shown to strongly inhibit bone nodule formation and mineralization of primary rat osteoblasts, without affecting the production of fibrous or soluble collagen (29, 30). Recent studies have shown that UTP inhibited osteogenic differentiation by activating P2Y2R in bone marrow-derived stromal cells *via* ERK1/2 pathway (31). Consistently, Orriss et al. showed increased bone formation by osteoblasts from P2Y2-KO mice which supported the notion of P2Y2R as a negative regulator of bone mineralization. They also found that defective resorption and decreased basal ATP release level in osteoclasts were present in P2Y2-KO mice (32). In contrast, a study using P2Y2R-KO mice on a different genetic background (SV129) showed an anti-osteogenic phenotype (34), whilst in a rat model, P2Y2R overexpression led to altered bone remodeling but mix phenotype, including enhanced bone length and strength but reduced trabecular thickness (53). Interestingly, this inconsistence was also evidenced in single nucleotide polymorphisms (SNPs) human cohort studies which clear associations between P2Y2R SNPs and bone mineral density (BMD) was established. In a Danish osteoporosis prevention study cohort, gain-of-function P2Y2R Arg312Ser SNP is correlated with higher BMD (54), while in a cohort of Dutch fracture patients, this correlation was not observed (55). Nevertheless, despite of these discrepancies, there is a consensus that the primary action of P2Y2R regulates mechanotransduction and extracellular ATP levels in bone. P2Y2R was first discovered to mediate the propagation of intercellular Ca^2+^ waves in osteoblasts in an autocrine manner after mechanical stimulation (33). Mechanistically, activation of P2Y2R in osteoblasts was found to sensitize mechanical stress-activated calcium influx as well as fast activation of multiple intracellular signaling pathways, including ERK1/2 and p38/MAPK, protein kinase C (PKC), RhoA GTPase and c-Jun N-terminal kinases (JNK1) (56–60). Using P2Y2R KO murine cells, Xing et al. demonstrated that KO-osteoblasts had reduced response, in terms of ERK1/2 phosphorylation, to both ATP and mechanical stimulation (34). In osteoclast, P2Y2R activation, whether acute or long-term, both promoted the release of ATP from osteoclasts, which indicates that the P2Y2R possibly regulate osteoclast function indirectly by promoting ATP release (32). In short, although the role of P2Y2R in bone homeostasis is possibly genetic background or species dependent, it has a primary role of regulating mechanotransduction and extracellular ATP release in bone cells.

Unlike P2Y2R, the molecular mechanisms of P2Y4R involvement in regulating bone cells are still unclear, despite both P2YRs are Gq/11 coupled receptors activated by UTP. It was only suggested that P2Y4R may play a ‘off-switch’ role during bone formation with redundancy of function of P2Y2R (35).

P2Y6R, which is selectively stimulated by UDP, has been found to be expressed in osteoclasts. UDP, released from mechanical stimulation or inflammation, can act through P2Y6R to induces the translocation and activation of NFκB in osteoclasts and their precursors, preventing apoptosis induced by TNFα pathway (37). To further investigate the role of P2Y6R in osteoclasts, Orriss et al. found functional defects in osteoclasts derived from P2Y6R-KO mice. The histomorphometric analysis showed that surfaces of endocortical and trabecular bone tissue occupied by osteoclasts were reduced in P2Y6R-KO mice (36). However, in another study, selective activation of P2Y6R by the UDP analog PSB0474 promoted the osteogenic differentiation from human bone marrow stromal cells (BMSCs) in postmenopausal women. After selectively blocking P2Y6R, decreased osteogenic differentiation were found at all culture time points. This suggests that targeting P2Y6R may cause harm to osteoblasts and subsequently lead to more bone loss in postmenopausal osteoporosis (38).

Currently, very limited studies have been done to investigate the involvement of P2Y11R in bone. P2Y11R has been so far identified to be expressed in human osteoclasts, but its molecular mechanism and functions are still not clear (61). More recently, P2Y11R has been found to be involved in breast cancer bone metastasis. Liu et al. suggested that ATP released from osteocytes hemichannels bound P2Y11R thereby suppressing the expression of chemokine receptor CXCR4, resulting in the inhibition of cell migration and bone metastasis of breast cancer (39). In fact, the most common skeletal complication of breast cancer is osteolytic bone metastasis mediated by osteoclasts (62). This could underscore the connection between P2Y11R’s expression in osteoclasts and the osteoclast-oriented formation of pre-metastasis niche and bone tropism of cancer cells, which warrant further investigation.

### P2Y12-Like Receptors

P2Y12-like receptors have long been in the focus of skeletal disease as they mediate more key regulatory genes in the context of bone. Abnormal function of P2Y12-like receptors causes distinct bone phenotype like bone loss.

The Gi/Go-coupled receptor P2Y12R has been found to be expressed in both osteoblasts and osteoclasts. Syberg et al. demonstrated that clopidogrel, an antagonist of P2Y12R, not only inhibited osteoblastogenesis, but also reduced the bone mass and strength by ~20% in mice (40). This was further confirmed in a patient cohort study in which treatment with clopidogrel led to a 60% increase in risk of fractures (41). However, conflicted results were shown by Su et al. In this study, mice treated with clopidogrel were protected from pathologic osteolysis. P2Y12R-KO mice were partially protected from age-related and pathological bone loss with reduced osteoclast function, suggesting a pro-resorption role of P2Y12R possibly *via* the Ras-related protein (RAP1) signaling (42, 63). Therefore, whether the predominant function of P2Y12R in bone is pro-formation or pro-resorption remains to be investigated.

As another Gi/Go-coupled receptor, P2Y13R has high affinity for ADP. In the past decades, more functions of P2Y13R in maintaining bone homeostasis have been gradually revealed, confirming its potential role in the process of fighting bone diseases. The expression of P2Y13R in osteoblasts and osteoclasts were firstly confirmed in 2010 (24). Its downstream signaling pathway involves Ras homolog gene family member A (RhoA)/Rho-associated and coiled-coil containing protein kinase I (ROCK I) signaling, which were inhibited after P2Y13 depletion and in turn reduced the MAPK/ERK signaling pathway and osteoblasts differentiation (43, 44). Consistent with these findings, Biver et al. showed that ADP stimulated the activity of transcription factor Runx2 *via* the RhoA/ROCK1 signaling pathway in a P2Y13R-dependent way, thereby stimulating the differentiation of pre-osteoblasts to osteoblasts. P2Y13R was proposed as the main receptor mediating the balance of osteoblast and adipocyte terminal differentiation from bone marrow progenitors (45). Further studies using P2Y13R KO mice demonstrated that P2Y13R deficiency in mature mice resulted in an abnormal bone phenotype, including less trabecular bone but thicker cortical bone. This was a consequence of reduced rates of bone turnover caused by decreased number and function of both osteoblasts and osteoclasts, confirmed by both *in vivo* and *in vitro* data (44). In addition, KO mice showed a higher osteogenic response against non-invasive axial mechanical loading due to the lack of a P2Y13R-regulated negative feedback pathway for ATP release, which was supported by *in vitro* evidence suggesting reduced extracellular ATP degradation by ALP in KO primary osteoblasts. More interestingly, KO mice also appeared to be protected from estrogen-deficiency induced bone loss (46). These findings offer an inspiring prospect for P2Y13R based osteoporosis therapy which may combine anti-P2Y13 receptor drugs with exercise to provided anti-resorptive and anabolic effects simultaneously. Furthermore, P2Y13R also plays a critical role in skeletal development *via* coordinating with hormonal regulators of phosphate homeostasis. Increased osteoblasts and decreased osteoclasts were observed in 4-weeks old young mice, whilst mature mice (>10 weeks old) showed an opposite phenotype. This age-dependent skeletal phenotype change has been considered to be related to higher serum fibroblast growth factor-23 (FGF-23) and phosphorus levels. Thus, P2Y13R were thought to regulate bone development in two different ways, which are the endocrine regulation of phosphate and FGF23 homeostasis at younger ages and the direct regulation of bone cells through bone remodeling at mature age (47). With evidence mentioned above, it is plausible that the P2Y13R can not only be considered as a potential pharmacological target for the treatment of osteoporosis, but also provide a promising target for treating phosphate metabolism-related bone diseases, including hypo- and hyperphosphatemia.

P2Y14R is the only P2Y receptor that can be stimulated by UDP-sugars but not ATP, UTP, and other naturally occurring diphosphate or triphosphate nucleotides (64). Evidence suggested that that extracellular UDP-sugars promoted expression of bone marrow receptor activator of NF-κB ligand (RANKL) and potentiated RANKL-induced osteoclastogenesis (48, 49). Lee et al. also found that the expression of P2Y14R can be selectively induced during RANKL-induced osteoclastogenesis both at the transcriptional and translational levels, while the protein expression of P2Y14R was downregulated when MAPK pathway was inhibited. More intriguingly, downregulation of P2Y14R by RNA interference (RNAi) was shown to suppress osteoclastogenesis (48). These data have clearly demonstrated a pro-osteoclastogenesis role for P2Y14R, whilst more recent evidence also suggested the involvement of P2Y14R in osteoblast biology. Using two murine osteoblast cell models, Mikolajewicz et al. found that P2Y14R negatively correlated with the efficiency of calcium signaling in response to mechanical and purinergic stimulation, but positively stimulated osteoblast proliferation possibly *via* modulating P2Y14-dependent ERK1/2 and AMPKα phosphorylation. Further data suggested that P2Y14R was also involved in osteogenic differentiation but its exact role needs further clarification (50). Although P2Y14R’s dual role in promoting osteoclast formation and osteoblast proliferation might dampen its potential as a therapeutic target for treating bone wasting diseases, its involvement in osteoblastic responsiveness to mechanical stimulation is still of great scientific interest and may offer a prospective opportunity in enhancing the anabolic effect of exercise in bone.

## Discussion

Although the molecular mechanisms of P2YRs’ involved in bone biology still need further elucidation, it is plausible that significant progress has been made in recent years. It is now clear that most of P2YRs play a dual role of both anabolic and catabolic functions during bone remodeling by affecting osteoblast and osteoclast simultaneously. To further fully understand the role of extracellular nucleotides-P2YRs signaling in the process of bone homeostasis, future research should elucidate the specific contribution of individual P2YRs in all types of bone cells including osteocytes and bone lining cells. No studies have yet revealed the expression of P2YRs in bone lining cells. Although P2Y2R, P2Y4R, P2Y6R, P2Y12R, and P2Y13R have been shown to be expressed in osteocytes, their roles in regulating the function of osteocytes are still unclear (65). Osteocytes are not only the most abundant type of bone cells but also the main mechanosensors within the bone. With their ligands-extracellular nucleotides being one of the main transduction signals of mechanical stimulation, several P2YRs, mainly P2Y2R, P2Y6R, P2Y13R, and P2Y14R, have being shown to be major players in regulating mechanotransduction. Therefore, further clarifying P2YRs’ roles in osteocytes and mechanotransduction will bring in potential scientific and clinical benefits, such as enhancing the anabolic effect of exercise in bone *via* targeting P2Y2Rs. Furthermore, the availability of global P2YR-KO rodent models and specific antagonists have provided us powerful tools in investigating P2YRs in bone. However, due to variable subtypes of P2YRs existing on bone cells and their complex cell/tissue/specie/age dependent functions, more specific research, such as examining the dynamic interaction and redundancy among all P2YRs and using tissue-specific conditional P2Y-KO murine model with higher physiological relevance, are needed in the future. In addition, more patient cohort studies, such as research in P2YR SNPs and osteoporosis/fracture risks, are also warranted to verify all pre-clinical findings in human settings before candidate P2YRs based targets enter the long and costly drug development pipelines. Finally, due to the nature of their ligands being extracellular nucleotides, which are keys to fundamental pathophysiological processes such as inflammation and cancer, P2YRs should be further researched in other bone diseases, particularly, osteoarthritis and cancer induced bone disease.

## Author Contributions

YZ: Conceptualization, Writing - Original Draft; HA: Visualization, Writing - Review & Editing; NW: Conceptualization, Supervision, Writing - Review & Editing. All authors contributed to the article and approved the submitted version.

## Conflict of Interest

The authors declare that the research was conducted in the absence of any commercial or financial relationships that could be construed as a potential conflict of interest.

## Publisher’s Note

All claims expressed in this article are solely those of the authors and do not necessarily represent those of their affiliated organizations, or those of the publisher, the editors and the reviewers. Any product that may be evaluated in this article, or claim that may be made by its manufacturer, is not guaranteed or endorsed by the publisher.
